# Glycosylation and Serological Reactivity of an Expression-enhanced SARS-CoV-2 Viral Spike Mimetic

**DOI:** 10.1016/j.jmb.2021.167332

**Published:** 2022-01-30

**Authors:** Himanshi Chawla, Sian E. Jossi, Sian E. Faustini, Firdaus Samsudin, Joel D. Allen, Yasunori Watanabe, Maddy L. Newby, Edith Marcial-Juárez, Rachel E. Lamerton, Jason S. McLellan, Peter J. Bond, Alex G. Richter, Adam F. Cunningham, Max Crispin

**Affiliations:** 1 School of Biological Sciences, University of Southampton, Southampton SO17 1BJ, UK; 2 Institute of Immunology and Immunotherapy, University of Birmingham, Birmingham B15 2TT, UK; 3 Bioinformatics Institute, Agency for Science, Technology and Research (A*STAR), Singapore 138671, Singapore; 4 Oxford Glycobiology Institute, Department of Biochemistry, University of Oxford, South Parks Road, Oxford OX1 3QU, UK; 5 Department of Molecular Biosciences, The University of Texas at Austin, Austin, TX 78712, USA; 6 Department of Biological Sciences, National University of Singapore, Singapore 117543, Singapore

**Keywords:** SARS-CoV-2, glycosylation, serology, glycoprotein, antibody

## Abstract

Extensive glycosylation of viral glycoproteins is a key feature of the antigenic surface of viruses and yet glycan processing can also be influenced by the manner of their recombinant production. The low yields of the soluble form of the trimeric spike (S) glycoprotein from SARS-CoV-2 has prompted advances in protein engineering that have greatly enhanced the stability and yields of the glycoprotein. The latest expression-enhanced version of the spike incorporates six proline substitutions to stabilize the prefusion conformation (termed SARS-CoV-2 S HexaPro). Although the substitutions greatly enhanced expression whilst not compromising protein structure, the influence of these substitutions on glycan processing has not been explored. Here, we show that the site-specific N-linked glycosylation of the expression-enhanced HexaPro resembles that of an earlier version containing two proline substitutions (2P), and that both capture features of native viral glycosylation. However, there are site-specific differences in glycosylation of HexaPro when compared to 2P. Despite these discrepancies, analysis of the serological reactivity of clinical samples from infected individuals confirmed that both HexaPro and 2P protein are equally able to detect IgG, IgA, and IgM responses in all sera analysed. Moreover, we extend this observation to include an analysis of glycan engineered S protein, whereby all N-linked glycans were converted to oligomannose-type and conclude that serological activity is not impacted by large scale changes in glycosylation. These observations suggest that variations in glycan processing will not impact the serological assessments currently being performed across the globe.

## Highlights

• HexaPro and 2P are recombinant glycoprotein versions of SARS-CoV-2 spike (S).
• HexaPro is an expression-enhanced version of SARS-CoV-2 S protein.
• Compared to 2P, HexaPro exhibits localised perturbations in glycosylation.
• Binding of antibodies from COVID-19 patients was insensitive to the glycoform of S.
• These results suggests that variations in S protein glycosylation will not impact serological studies.

## Graphical abstract

**Figure:**
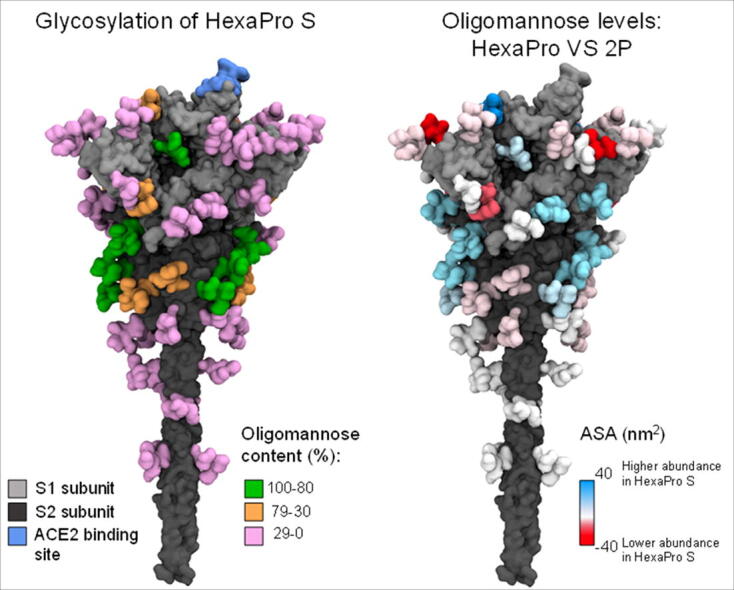

## Introduction

Recombinant viral glycoproteins are an important resource for vaccine development, diagnostics and as research reagents. Viral glycoprotein glycosylation can influence an extensive range of properties including immunogen trafficking,^1^ immunogenicity,^2^, ^3^ antigenicity^4^, ^5^ and serum clearance rates^6^. Importantly, recombinant viral spike glycosylation can be influenced both by features of the glycoprotein sequence, such as glycan density and protein architecture^7^, ^8^, ^9^ and an array of expression conditions, such as producer cell type and expression conditions.^10^, ^11^, ^12^, ^13^ It is therefore important to define the glycosylation of recombinant viral glycoproteins and monitor changes that may occur during target optimization and the development of manufacturing procedures.^14^, ^15^ As carbohydrates on viral proteins can influence the immune response, it is important to look at the binding of sera antibodies to antigens possessing distinct glycoforms.^16^ Here, we investigate the antigenic properties of glycoprotein reagents developed in response to the coronavirus infectious disease 2019 (COVID-19) pandemic, focused on the viral S glycoprotein.^17^, ^18^

The causative agent of COVID-19, Severe acute respiratory syndrome coronavirus-2 (SARS-CoV-2), is a positive-sense single-stranded RNA virus that has caused significant morbidity and mortality throughout the world.^19^, ^20^ Like other coronaviruses, SARS-CoV-2 utilizes the S glycoprotein for recognition of the host cell entry receptor and subsequent membrane fusion, which is mediated by the S1 and S2 subunits, respectively.^21^ The S protein is a trimeric class I fusion protein and is post-translationally cleaved into S1 and S2 subunits using the host cellular protease, furin.^22^ The S1 subunit possesses an N-terminal domain (NTD) and a receptor-binding domain (RBD), also known as domain A and B, respectively.^23^

The exposed position of the RBD enables binding to the angiotensin converting enzyme 2 (ACE2) receptor^24^ and, as a result, it is the main target of anti-SARS antibodies during infection.^25^, ^26^, ^27^, ^28^, ^29^, ^30^ Due to this phenomenon, combined with its high recombinant protein yields, several antibody tests have been developed using RBD as a tool to test for previous SARS-CoV-2 infection.^31^, ^32^, ^33^, ^34^ One disadvantage of using RBD as an antigenic bait for testing is that it may not capture the entire antibody response to the S protein as it lacks the full trimeric structure.^35^ In addition to RBD, other antibody tests use the nucleoprotein (N protein) as antigenic bait to detect prior SARS-CoV-2 infection, such as the Abbott test.^36^, ^37^ Similarly to the RBD, N protein will not capture the complete antigenic surface and therefore may not reveal the full immune response to SARS-CoV-2 infection. As the S protein is the prime target of neutralizing antibodies, the native-like trimeric S glycoprotein may facilitate the presentation of a more complete range of epitopes for antibody testing.^38^, ^39^ Serological testing requires that the protein is both stable and that production is readily scalable for widespread use. There has been significant development in design of S protein constructs to facilitate increased recombinant production and protein stability.

**Figure 1:**
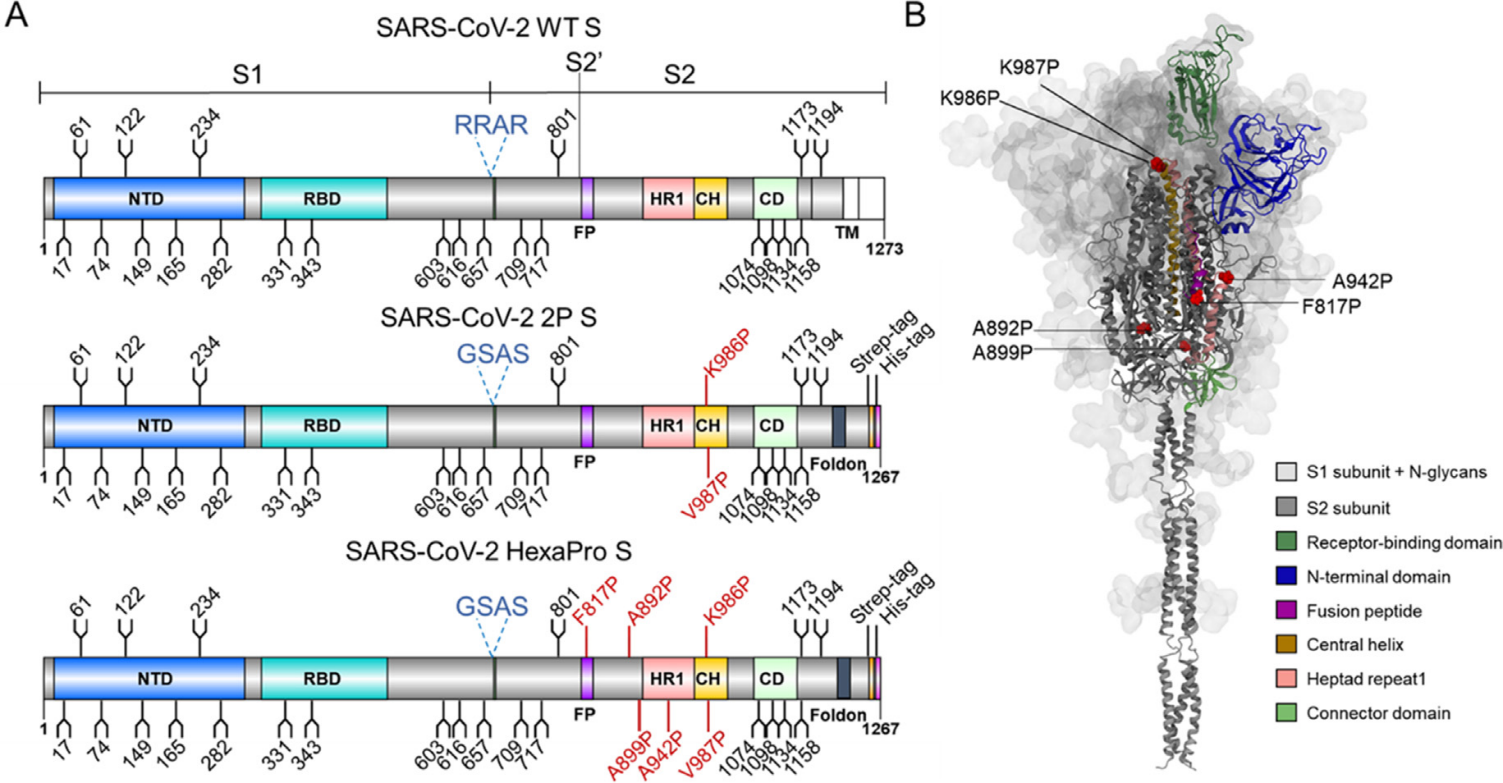
**Representation and characterization of recombinant SARS-CoV-2 spike protein**. (A) The protein domains are represented as N-terminal domain (NTD), receptor-binding domain (RBD), fusion peptide (FP), heptad repeat 1 (HR1), central helix (CH), connector domain (CD), and transmembrane domain (TM). The fusion cleavage site is illustrated as dashed lines (blue). N-linked glycosylation sequons (N-X-S/T, where X ≠ P) are shown as branches. SARS-CoV-2 WT presents S1 and S2 domain with furin cleavage site (RRAR) and transmembrane domain at C-terminal end. SARS-CoV-2 2P prefusion stabilized protein with proline substitutions at residues 986 and 987 and, a “GSAS” mutation at furin cleavage site (residues 682–685). HexaPro prefusion stabilized protein of SARS-CoV-2 with a “GSAS” mutation at furin cleavage site and six proline substitutions, highlighted in red. (B) Structural representation of HexaPro S protein illustrating six proline substitutions (red spheres) in SARS-CoV-2 ectodomain (PDB ID: 6XKL). The S1 subunit along with N-glycans are shown as transparent molecular surface. The S2 subunit is shown in dark grey. Different domains present in S1/S2 subunits are highlighted in respective colors in ribbon diagram of only one protomer.

Prefusion stabilization strategies have been employed for class I fusion proteins to increase the recombinant expression of viral glycoproteins.^40^, ^41^, ^42^, ^43^, ^44^ A common strategy is the introduction of proline substitutions which impedes the switch to the post fusion conformation.^45^ This is crucial as neutralizing antibody epitopes are predominantly presented on the prefusion conformation.^41^, ^46^, ^47^, ^48^ For SARS-CoV-2, the expression of a stable, soluble form of the S-protein was originally achieved by truncation at the transmembrane domain and the incorporation of two proline residues (K986P and V987P)^17^ (Figure 1; SARS-CoV-2 2P S, henceforth termed “2P”). Despite the utility of the 2P construct for structural analysis^17^ and serological testing,^35^, ^49^, ^50^ the low expression levels prompted the development of an expression enhanced version containing four additional prolines (Figure 1; SARS-CoV-2 HexaPro, henceforth termed “HexaPro”).^18^ HexaPro exhibits native-like protein architecture, antigenic properties, and contains the twenty-two N-linked glycosylation sites of the native viral spike.^18^, ^51^, ^52^ Additionally, HexaPro has shown promising results as a vaccine candidate in mice immunization, resulting in high-titre neutralizing antibodies.^53^, ^54^, ^55^

Here, we performed Liquid chromatography-mass spectrometry (LC-MS) experiments to establish whether the additional stabilizing mutations impact the presentation of glycans on the surface of the S protein. Further, to explore whether the observed differences in glycans impacted the HexaPro protein function, we compared the binding of HexaPro and 2P protein to ACE2 using surface plasmon resonance (SPR) and demonstrated comparable binding between the two. Molecular dynamics (MD) simulations revealed a similar pattern of surface accessibility of N-linked glycan sites between the 2P and HexaPro S proteins, and support the overall conserved nature of glycosylation. In addition, we explored whether the modest changes in glycosylation impacted the detection of immune responses in patient sera previously infected with COVID-19. Both HexaPro and 2P protein were successful at detecting an immune response towards SARS-CoV-2 in both hospitalized and non-hospitalized patients. Furthermore, we compared the binding of S protein possessing oligomannose-type glycans at all potential N-linked glycosylation sites (PNGS), which was achieved through co-transfection with the ER α mannosidase I inhibitor, kifunensine. Both S protein glycan variants revealed highly similar binding to sera from patients with prior-SARS-CoV-2 infection. These studies further support the use of hyperstabilization using additional proline mutations and demonstrate its utility in serological testing.

## Results and discussion

### Characterization of prefusion stabilized SARS-CoV-2 HexaPro S protein

**Figure 2:**
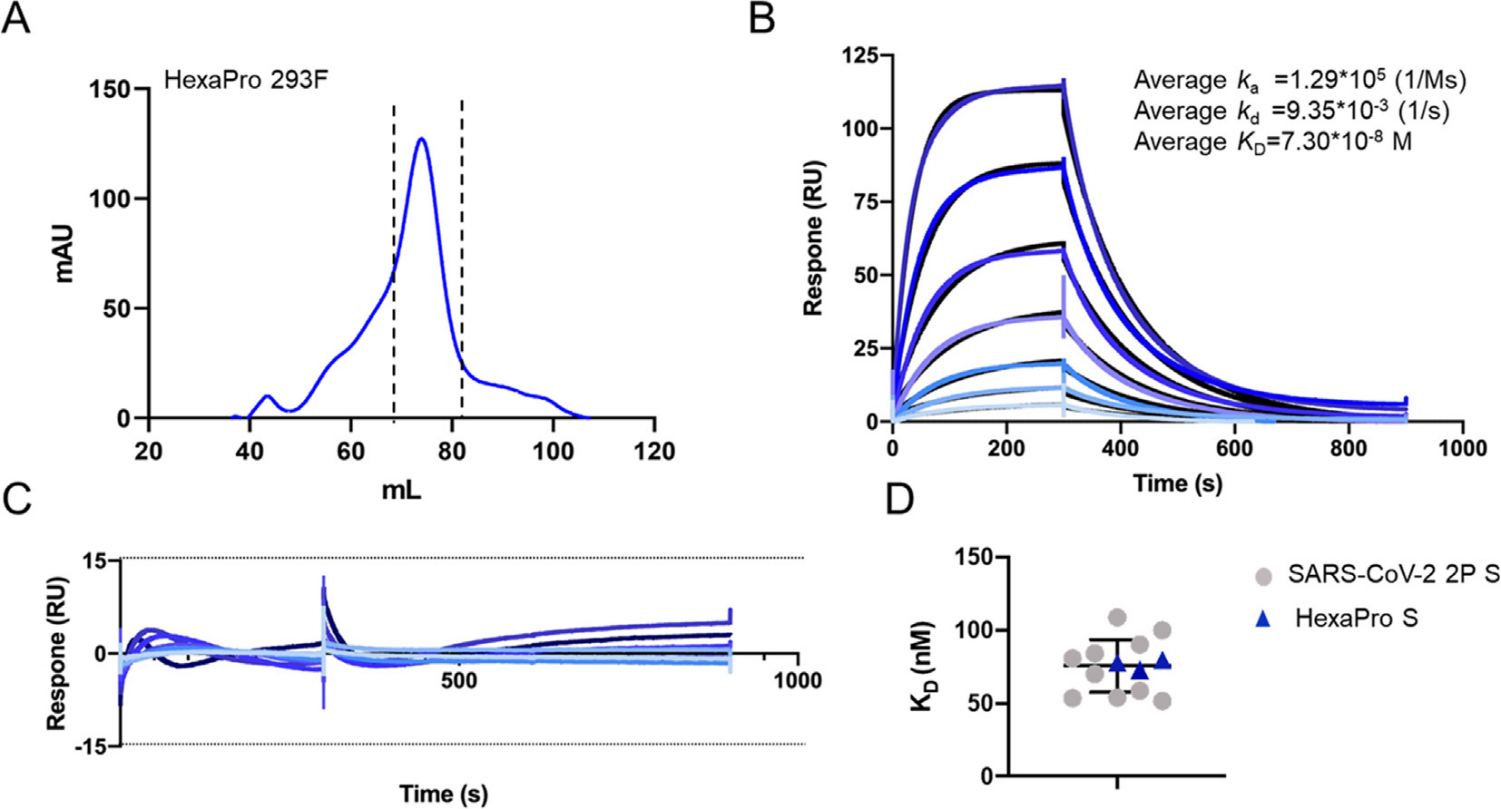
**Characterization of recombinant SARS-COV-2 S protein, HexaPro.** (A) SEC of affinity-purified recombinantly expressed S protein. Dashed lines indicate fractions collected for subsequent use. (B) SPR of HexaPro (ligand) with soluble ACE2 receptor (analyte). Dark blue to light blue lines represent the serial dilutions of ACE2 protein from 200 nM to 3.125 nM, respectively. Black lines are fitted values of the respective concentration to illustrate the best fit to a 1:1 binding model. Three repeats were performed and averaged to determine the *k*_a_*_,_ k*_d_ and *K*_D_ values. (C) Residual plot illustrating the deviation of the fitted data to the raw values of the experimental data at different concentrations. (D) Representation of *K*_D_ values determined using various repeats, grey dots illustrate the binding of 2P with ACE2 (values reproduced from 56) and the binding of HexaPro with ACE2 are shown as blue triangles. The mean of *K*_D_ values of 2P is plotted as a black line and the error bars represent ±standard deviation calculated using GraphPad Prism.

For characterization of prefusion stabilized SARS-CoV-2 S protein, we transiently transfected plasmid encoding SARS-CoV-2 S protein containing the HexaPro stabilizing mutations in Human embryonic kidney (HEK) 293F cells. To ensure the analysis of only native-like trimeric protein, the supernatant was first purified using nickel-affinity chromatography followed by size exclusion chromatography (SEC) (Figure 2(A)). To functionally characterize the binding of the expressed protein with ACE-2, the binding affinity (expressed here using the dissociation constant, *K*_D_) of HexaPro with a soluble recombinant ACE2 was determined using surface plasmon resonance (SPR) which was repeated three times to determine the average *K*_D_ (Figure 2(B)). A residual plot of the data revealed minimal deviation between observed values and calculated values using a 1:1 binding model between SARS-CoV-2 HexaPro S protein and ACE2 (Figure 2(C)). The *K*_D_ values are comparable to that previously reported for 2P^56^ (Figure 2(D)).

### Determination of impact of proline mutations on spike glycosylation

**Figure 3:**
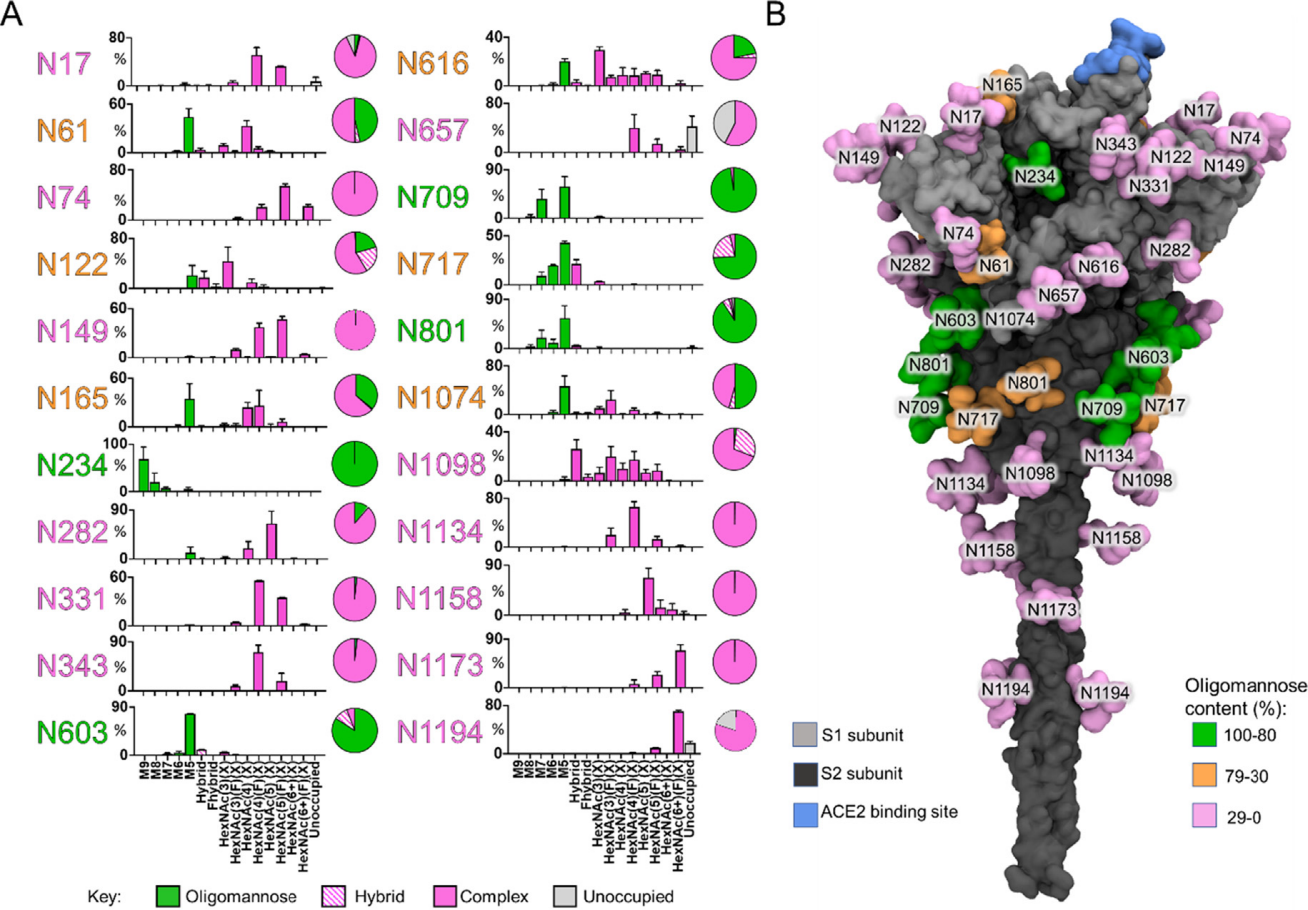
**Site-specific glycosylation of expression-enhanced recombinant trimer of SARS-CoV-2 S protein (HexaPro).** (A) Relative quantification of the N-linked glycosylation sites of trimeric S protein, produced in HEK293F cells. The bar graph represents the mean of three independently expressed replicates with error bars representing the standard error of the mean. The color codes in the schematic illustrates the processing state of glycans from least processed to most processed, oligomannose (green), hybrid (dashed pink), and complex glycans (pink). The proportion of unoccupied N-linked glycan sites are represented in grey. The pie charts summarize the quantification of these categories. The N-linked glycan site labels are colored based on the oligomannose-type glycan content, green (80–100%), orange (30–79%) and magenta (0–29%). (B) The model was constructed using the prefusion structure of trimeric SARS-CoV-2 S glycoprotein as described in Materials and Methods. The S1 and S2 subunits are shown as light and dark grey, respectively. The glycans sites are categorized as high-mannose type glycans (green), hybrid glycans (orange), and complex-type glycans (pink). The ACE2 receptor binding site is shown in blue.

To determine the effect of stabilizing mutations on glycosylation, we determined the site-specific glycan compositions of SARS-CoV-2 HexaPro. In preparation for glycopeptide analysis, we independently expressed and purified three replicates of the soluble ectodomain of the SARS-CoV-2 protein truncated at the transmembrane domain. We note that this construct was used to determine the high resolution cryo-electron microscopy (cryo-EM) structure.^18^ To generate glycopeptide samples derived from these batches of protein, we employed three different protease enzymes, trypsin, chymotrypsin, and alpha-lytic protease. These proteases cleave the protein chain at specific amino acids and were selected to generate glycopeptides that contain a single glycosylation sequon (N-X-S/T-X, where X-any amino acid except proline). Using liquid chromatography-electrospray ionization mass spectrometry (LC-ESI MS), we were able to quantify the oligomannose-type glycans, complex-type glycans and the proportion of unoccupied potential N-glycosylation sites (PNGS) across all 22 N-linked glycan sites on the HexaPro protomers (Figure 3(A and B)).

**Figure 4:**
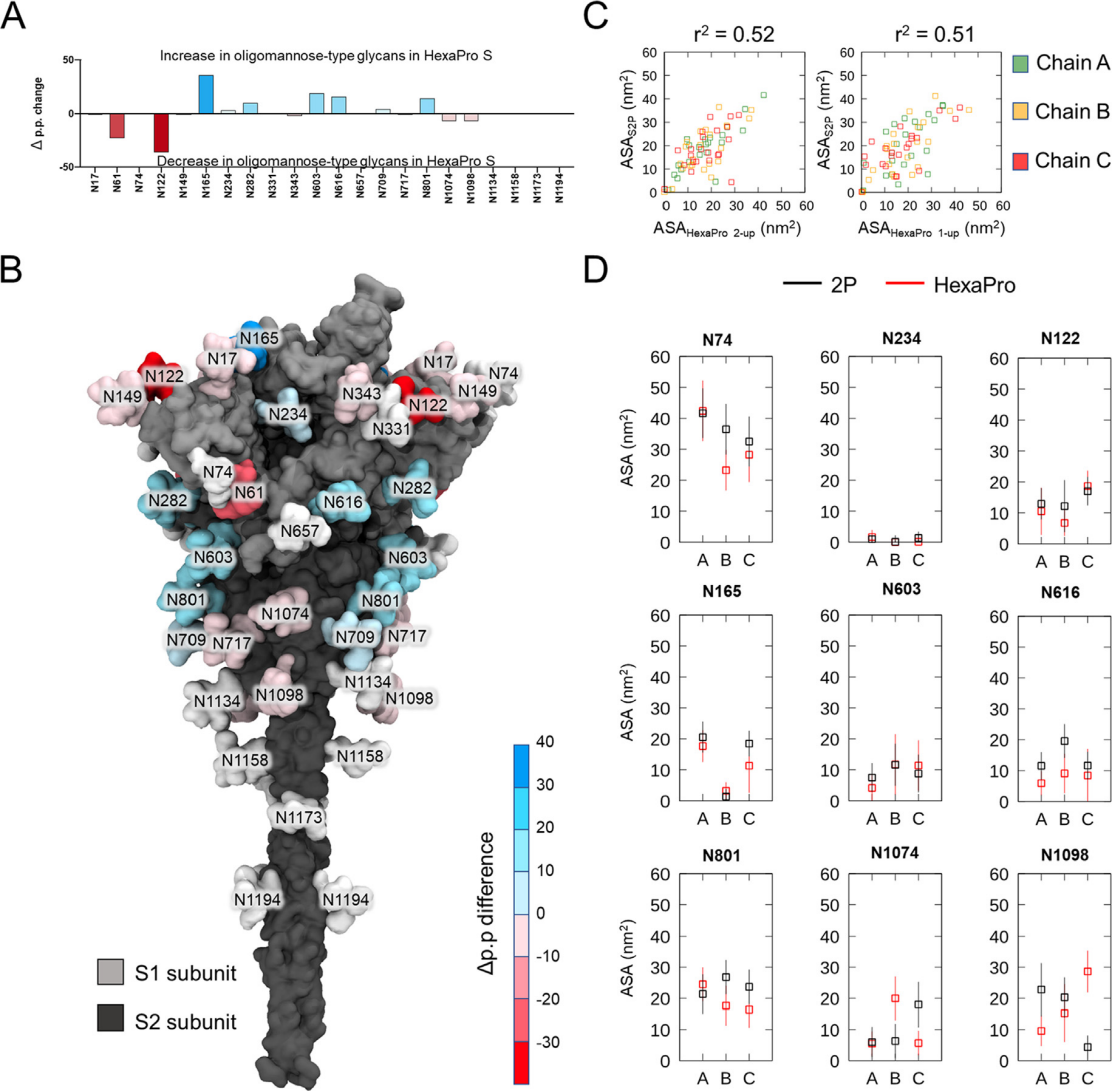
**Comparison of glycan composition across prefusion-stabilized SARS-CoV-2 S protein.** (A) The percentage point change in oligomannose-type glycan content between SARS-CoV-2 S protein, HexaPro and 2P. The percentage point (p.p.) difference on the y-axis is the arithmetic difference between the percentiles of oligomannose-type glycans between the two populations (here defined as: p.p. = % HexaPro – % 2P). Positive values (blue) indicate a higher abundance of oligomannose-type glycans in HexaPro relative to 2P. Negative values (red) indicate a lower abundance of oligomannose-type glycans in HexaPro relative to 2P. (B) A full length model of SARS-CoV-2 S protein with N-glycans colored based on the percentage point change values. The scale represents the differences in oligomannose-type glycans observed in HexaPro when compared to 2P protein. Colors correspond to p.p. values in Panel A. The model was constructed using prefusion structure of trimeric SARS-CoV-2 S glycoprotein, as detailed in Materials and Methods. (C) Correlation of ASA values between HexaPro and 2P S protein. The average ASA values of all glycans from three replica simulations of two-RBD-up HexaPro (left side) and one-RBD-up (right side) structures plotted against the average ASA values from simulations of the respective 2P structures. (D) The average ASA values for 2P (black) and HexaPro two-RBD-up (red) with error bars showing standard deviations along the trajectories and across three repeat simulations. The displayed sites are those with changes in the oligomannose content across both versions. N74 (high ASA values) and N234 (lowest ASA values) were used as a reference. The chain (A, B, or C) of the trimeric S protein is indicated along the x-axis.

To determine the potential impact of more extensive modifications on the prefusion stabilized S protein, we compared the glycan profile of SARS-CoV-2 S protein of 2P with HexaPro. Interestingly, the overall processing states of the recombinant S protein were conserved across both the versions, with few variations at the site-specific level (Figures 3(B) and 4(A)). Site-specific analysis of these recombinant proteins suggests that the levels of oligomannose-type glycans are consistent with native S protein on infectious virus and also with other coronaviruses.^51^, ^52^, ^57^, ^58^, ^59^

In addition to the information obtained from studying the populations of oligomannose-type glycans at individual glycosylation sites, understanding the processing of complex-type glycans is also informative when considering immunogen design, reagents for serological studies, and for understanding the extent to which recombinant glycoprotein can be used as mimics of the functional viral spike. For example, the epitope of neutralizing antibody S309, which targets the S protein of SARS-CoV-1 and SARS-CoV-2, contains a fucosylated glycans at N343.^60^ HexaPro is 99% fucosylated at the N343 site, with almost all the glycans bearing fucose residues (Supplementary Table S1). Furthermore, sulfated N-linked glycans have been detected on viral glycoproteins and they could potentially play role in immune regulation, as in influenza.^61^, ^62^ We detected sulfation at several N-glycosylation sites on HexaPro (N74, N149, N1194) and observed a similar abundance of sulfation across both S proteins (2P and HexaPro), which is in accordance with analysis of other SARS-CoV-2 S proteins^63^, ^64^ (Supplementary Figure S1).

In all formats of SARS-CoV-2 S expressed in mammalian cells, a higher proportion of complex-type glycans compared to oligomannose-type glycans were observed.^51^, ^52^, ^59^, ^61^, ^63^, ^65^ Moreover, the complex-type glycans somewhat obscure immunogenic surfaces and constitute a shield to evade the immune system,^66^, ^67^, ^68^ albeit not at a level observed in many other viral envelopes.^21^, ^57^ Out of 22 N-linked glycan sites on each protomer, HexaPro S presents more than 50% highly processed complex-type glycans on 15 N-linked sites which is comparable to the 2P expressed in different laboratories.^51^, ^52^ The underoccupancy at the glycosylation sequon at N657 is present on both HexaPro (Figure 3(A) and Supplementary Table S1) and 2P (Supplementary Table S2). The glycan site at the C terminus, N1194, is fully occupied in the case of 2P (Supplementary Table S2) however, there is an elevation of unoccupied PNGS (18%) at N1194 on the HexaPro S protein (Supplementary Table S1).

The oligomannose-type glycan content of the glycans of the HexaPro protein (29%) (Supplementary Table S1) is lower when compared to other viral glycoproteins including HIV-1 Env (60%) and LASV GPC (49%).^69^, ^70^ This is consistent with earlier observations using 2P protein which indicated that SARS-CoV-2 S is less shielded, which may be beneficial for the elicitation of neutralizing antibodies.^51^ Overall, there is a similar level of oligomannose-type glycans across both 2P and HexaPro (Supplementary Table S3).

Thus we have shown here that the site-specific glycosylation of the expression enhanced version of SARS-CoV-2, HexaPro, is highly similar to the glycosylation of 2P and native S protein as presented on the virus.^51^, ^52^, ^58^ Also, we confirm earlier observations^18^ that both forms of the recombinant protein have indistinguishable binding properties to the receptor, ACE2, indicating the functional form of protein is intact. However, we also detected some difference in glycosylation which could suggest differences in the conformational properties between the HexaPro and 2P. This motivated us to extend the analysis of protein conformational flexibility by performing MD simulations, and to characterize its functional behaviour by performing serological testing. This builds upon previous observations of cryo-EM and a small-scale serological evaluation.^18^

### Differences in oligomannose content between 2P and HexaPro

The glycan at a structural site, N234, which has been shown to stabilize the RBD up conformation in the trimeric structure of the S protein,^71^ is principally oligomannose-type, and is conserved across both the constructs of recombinant S protein (Figure 3(A and B)). Also, the oligomannose-type glycans of N234 likely arises from steric clashes with the protein component, which in turn limits the ability of glycan processing enzymes to act, as it is sandwiched between N-terminal and receptor-binding domains^71^ (Figure 4(B)). Overall, the oligomannose content is highly similar across both 2P and HexaPro (Supplementary Table S3). However, the Man_9_GlcNAc_2_ content is higher in the case of HexaPro compared to 2P, indicating the reduced accessibility to glycan processing enzymes^51^ (Supplementary Table S3). This could possibly be explained by the two RBD “up” conformation observed in HexaPro.^18^ Also, at the site-specific level there are several other sites which showed changes in oligomannose content across 2P and HexaPro (Figure 4(A and B)). Differences in glycan processing states were observed at glycan sites N61, N122, N165, N603, N616 and N801. The major differences were observed at N165 and N122, which are in close proximity to the RBD (a roughly 50 percentage point difference in both cases). The N165 site possesses a higher abundance of oligomannose-type glycans on HexaPro shown in dark blue whereas the N122 site possesses a lower abundance of oligomannose-type glycans on HexaPro shown in red (Figure 4(B)).

To investigate the molecular basis of the observed changes in oligomannose-type glycan content across both the S proteins, we performed a series of triplicate 200 ns MD simulations of the HexaPro two-RBD-up, HexaPro one-RBD-up and 2P one-RBD-up constructs. The HexaPro two RBD-up model was generated by fitting the structure of HexaPro one-RBD-up to the electron density map of HexaPro two-RBD-up using molecular dynamics flexible fitting (MDFF) (details in Methods section). All potential N-linked glycosylation sites were glycosylated with Man_9_GlcNAc_2_ glycans, as these represent the primary substrate for glycan processing enzymes and hence may be used to predict glycan processing as previously described.^52^

First, we investigated the correlation between accessible surface area (ASA) for all glycans between HexaPro and 2P. There is a moderate correlation (r^2^ = 0.5) between both one-RBD-up and two-RBD-up HexaPro when compared to the 2P, suggesting there is some degree of difference in glycan accessibility between HexaPro (two-RBD-up & one-RBD-up) and 2P (one-RBD-up) (Figure 4(C)). To understand whether this difference is due to the protein architecture, or due to the stochastic nature of the simulations, we then compared the ASA for each of the glycosylation site between HexaPro and 2P. Furthermore, we calculated the arithmetic difference between the ASA values of HexaPro (two RBD-up) and 2P (one RBD-up) at each glycan site in all three chains (Supplementary Figure S2). The error bars represent standard deviations throughout the trajectories which capture the variation caused by sampling. A positive value represents lower accessibility in HexaPro, which could correlate to reduced glycan processing and a higher abundance of oligomannose-type glycans. The difference at most of the sites was small and with substantial standard deviations, suggesting that most of the differences arise from the stochastic sampling of the glycans.

Then, we further explored sites which showed changes in oligomannose content in the site-specific glycan analysis. For example, on HexaPro, N165 displays an increase of almost 50 percentage point in oligomannose-type glycan compositions when compared with 2P (Figure 4(A)). As the N165 site is in close proximity to the RBD region, its glycan processing state may be influenced by the orientation of the RBD. When comparing the model generated with two RBD domains in the up configuration, to one-RBD-up, the steric environment of the N165 and N122 sites were expected to change. However, the simulations comparing HexaPro two-RBD-up versus one-RBD-up showed little changes in solvent accessibility (Figure 4(D)). This is likely because in the HexaPro (two-RBD-up) simulation, the additional RBD in the initial up configuration tends to revert to a down state, and the final snapshot in all simulation replicas is similar to the 2P (one-RBD-up) simulation (Supplementary Figure S3). It is noteworthy that in our initial model, derived from the experimental electron density map of the HexaPro two RBD-up conformation, the RBD in chain C resembles an intermediate state between the up and down states, rather than a fully open conformation. The changes in ASA values we observed are only significant at a few sites and minor with variations across the three chains, indicating that the differences are due to sampling.

Finally, we performed protein-structural analyses to compare the dynamics of 2P and HexaPro. We observed similar root-mean square fluctuations (RMSF) profiles between the HexaPro (two-RBD-up and one-RBD-up) and 2P (one-RBD-up) simulations, except for the RBD in chain C, due to the up to down conformational changes described above (Supplementary Figure S4(A)). The principal motion measured during the simulations also reveals similar dynamics between these two S proteins (Supplementary Figure S4(B)). Hence, the simulations suggest that despite local glycan perturbation, the two S protein versions have very similar dynamics and our computational analysis does not provide evidence to support a steric explanation for the differences in glycosylation.

### Conservation of serological reactivity across recombinant SARS-CoV-2 S protein

**Figure 5:**
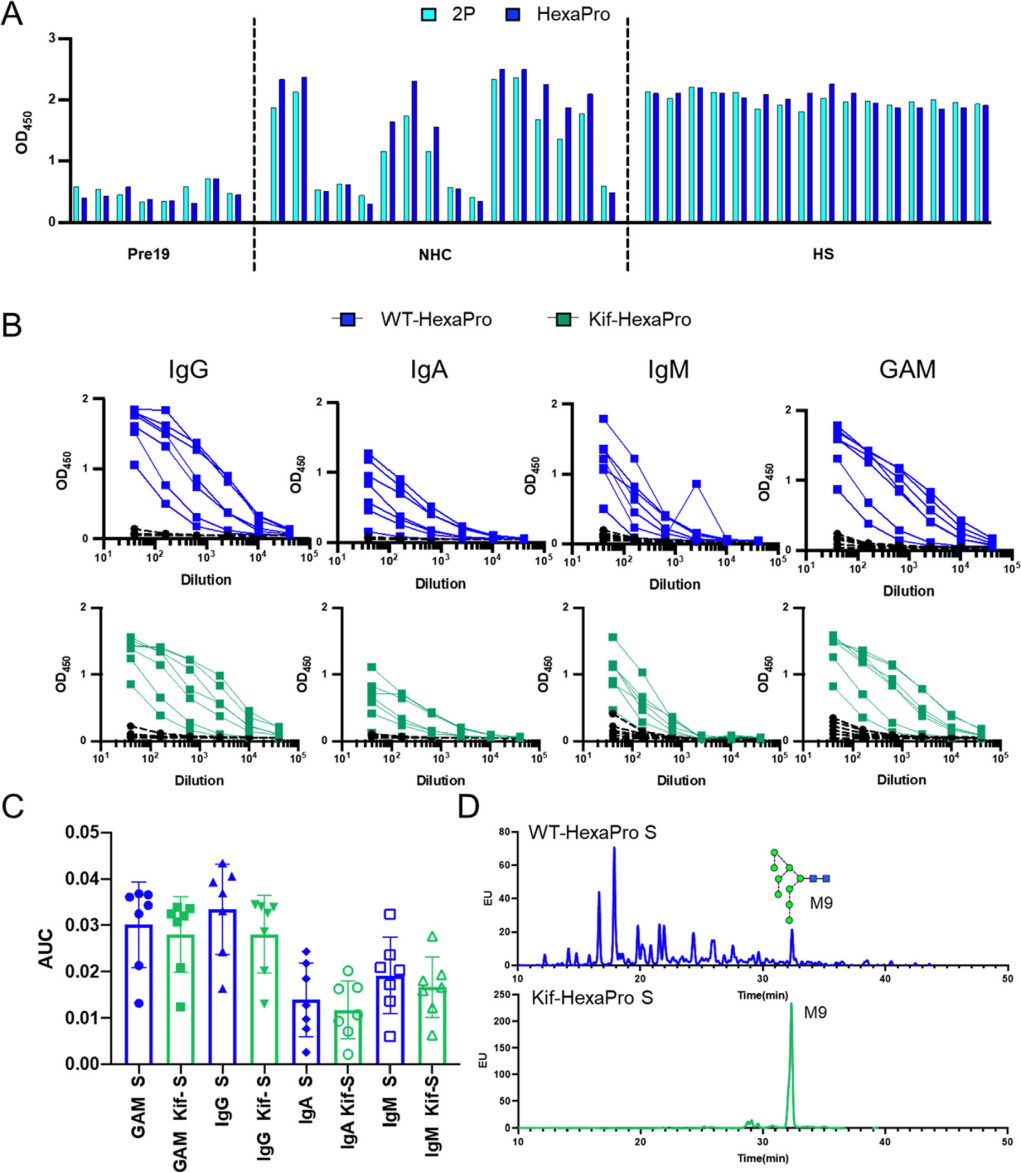
**Antibody binding to spike glycoproteins.** Individual serological responses from pre-2019 donors (Pre19, *n* = 8), non-hospitalized convalescent donors (NHC, *n* = 16) or PCR + hospitalized subjects (HS, *n* = 16) as determined by ELISA using HRP-labelled combined anti-IgG, IgA and IgM. A) Absorbance values of sera serially diluted from a starting dilution of 1:40 against 0.1 µg 2P (cyan bars) or HexaPro (blue bars). B) Absorbance values of sera serially diluted from a starting dilution of 1:40 from Pre-19 (black circle, dashed lines), NHC of HexaPro protein (blue squares) and Kifunensine-treated HexaPro (green squares) as determined by ELISA using HRP-labelled combined anti-IgG, IgA, IgM, and GAM. C) Area Under the Curve (AUC) of responses shown in figure B. The blue bars representing the AUC of HexaPro with IgG, IgA, IgM and GAM. The green bars representing the AUC of kif-treated HexaPro with different immunoglobulins. The mean ± standard deviation from the mean (SD) is plotted. D) HILIC-UPLC profile of N-linked glycans from WT (wildtype) HexaPro and Kifunensine-treated HexaPro produced in HEK 293F cells and purified by Ni^+2^ column followed by SEC. The blue peaks representing glycan spectra of WT-HexaPro. The green peaks representing glycan spectra of kifunensine-treated HexaPro showing only Man_9_GlcNAc_2_ (M9) glycans.

To compare the serological reactivity of the recombinant 2P and HexaPro S protein, we tested the binding of different immunoglobulin isotypes in sera from subjects with or without a prior SARS-CoV-2 infection to these viral antigens. This extends the observations presented by Hsieh et al. by using a larger and geographically distinct donor group and by examining a range of antibody isotypes.^18^ Sera from three groups of subjects from the United Kingdom were analyzed: hospitalized subjects (HS) which included individuals that were admitted to hospital and had RT-PCR confirmed SARS-CoV-2 infection; non-hospitalized convalescent (NHC) subjects, who were tested positive by clinically validated antibody test^35^ and were not hospitalized and a negative control group, from whom sera was taken before 2019 (Pre-19). As expected, strong IgG, IgA and IgM responses were detected to both S glycoproteins in all hospitalized subjects with severe disease (Figure 5(A) and Supplementary Figure S5(A)). In contrast to the strong responses observed in severe cases, IgG, IgA and IgM responses were observed in the NHC subjects, and in some instances these responses were not above those of control sera (Figure 5(A)). There was minimal binding of IgG to S glycoprotein by Pre19 sera. Both 2P and HexaPro showed comparable serological reactivity, with a slightly increased level of binding of patient, but not control, sera to HexaPro in the NHC sera (Figure 5(A)). Overall, the signal: noise ratio was superior for HexaPro compared to 2P, particularly as sera were diluted (Supplementary Figure S5(B)), but overall, a key conclusion is that the HexaPro was not inferior to the 2P glycoprotein.

Although the small difference in glycosylation between 2P and HexaPro did not result in diminished antibody binding to HexaPro, it is conceivable that wider batch-to-batch variations could impact consistency of serological reactivity. We therefore tested sera for antibody binding to glycoprotein with substantially engineered glycosylation. We expressed HexaPro in the presence of kifunensine (Kif) which results in oligomannose-type glycans and we confirmed the altered processing state using hydrophilic interaction ultra-performance liquid chromatography (HILIC-UPLC) analysis of fluorescently labelled pool of N-linked glycans (Figure 5(D)). The WT HexaPro chromatogram shows the presence of diverse glycans in contrast to Kif-treated HexaPro, where Man_9_GlcNAc_2_ (M9) glycans were predominant (Figure 5(D)). We compared IgG, IgA, IgM and combined IgGAM antibody binding to HexaPro with no glycan engineering (which we term ‘wildtype’) with Kif-treated HexaPro. The serological response detected using SARS-CoV-2 positive sera were similar whether kif-treated HexaPro or WT HexaPro was used in the assay (Figure 5(B)). Negligible binding was observed with pre-19 sera (shown in black) with both kif-treated and WT HexaPro. Area Under the Curve (AUC) calculations confirmed that both WT S and Kif-treated S protein were bound similarly by IgG, IgA, IgM and IgGAM (Figure 5(C)). This suggests that the immune response elicited following SARS-CoV-2 infection, with respect to immunoglobulin binding, is not dictated by the glycan processing state of the S protein, as converting the glycans at every site from their native-like compositions, does not impact the detection sensitivity.

## Perspectives

In this study we aimed to investigate a range of biophysical, glycan composition and serological binding properties of HexaPro, an expression-enhanced version of SARS-CoV-2 S protein. This version contains six proline mutations which lead to its high expression and stabilization, and promising immunogenic properties.^18^, ^53^, ^54^, ^55^, ^72^ We reveal the comparable affinity of HexaPro to the ACE2 receptor with the earlier version, 2P which has been used in several vaccine studies,^73^, ^74^, ^75^, ^76^ and extensively as a serological reagent.^35^, ^49^, ^77^ Furthermore, we determined the impact of these additional proline mutations on glycan composition using LC-ESI MS and compared it with the 2P. It was interesting to note that overall, the glycosylation was highly similar between both versions, except at a few sites. To further explore phenomena that could be directing these changes we performed MD simulations to decipher the conformational properties of both versions.

The structural site, N234 showed fully oligomannose-type glycans in both the versions suggesting that the central integrity of the protein architecture is unperturbed.^52^, ^58^, ^71^ Overall, the oligomannose content was similar in both the S proteins, however, the Man_9_GlcNAc_2_ content was higher in HexaPro, suggesting less accessibility to glycan processing enzymes. This motivated us to look at the accessible surface area (ASA) of oligomannose-type glycans in 2P and HexaPro using MD simulations. We did not see much difference in ASA values at N-glycan sites which showed changes in oligomannose-content determined using LC-MS. Also, we observed highly similar protein dynamics in both versions. This suggests that both of the proteins have very similar conformational properties. However, the simulations sample a short timescale while glycans are processed over a much longer timescale and these simulations do not provide evidence for the steric changes predicted from the differential glycosylation observed in the LC-MS data. It is conceivable that the changes in oligomannose-type glycan content could instead be due to high expression of HexaPro resulting in changes in productive enzyme:substrate recognition events. Overall, however, these results suggest that there are limited structural and dynamic differences between 2P and HexaPro.

S proteins are being deployed in serological testing and have proven to be effective in confirming prior infection of SARS-CoV-2 in infected patients.^35^, ^78^, ^79^ Due to its high expression, HexaPro could widen the availability of S protein for serological testing. In this study, we aimed to investigate the binding of HexaPro with sera IgG, IgA, and IgM to better understand its interaction with antibodies induced in COVID patients, in order to maximize the potential of HexaPro in these applications. We also see highly similar antibody binding with both 2P and HexaPro, suggesting that incorporation of mutations in HexaPro does not seem to affect immune recognition. Our results also reveal highly similar reactivity with glycoengineered HexaPro possessing all oligomannose-type glycans at all PNGS, indicating that sera binding is not readily impacted by the fine processing of the glycans of the S protein. Moreover, since the level of antibody binding was not significantly reduced after glycan engineering of HexaPro, the data may be interpreted as indicating that after natural infection most antibodies do not target epitopes that can be influenced by variations in glycan processing. If so, since antibodies from infected individuals can neutralise infection in vitro, it suggests that protection from infection is not associated with the targeting the terminal region of glycans. We note however that the predominant glycan at N234 is not changed by kifunensine. Overall, these observations suggest that variations in the S protein glycosylation of SARS-CoV-2 will not impact the serological assessments currently being performed across the globe.

## Materials and Methods

### Protein expression and purification

For expression of prefusion S ectodomain of SARS-CoV-2 HexaPro construct, the base construct of SARS-CoV-2 S 2P (GenBank: MN908947) having proline substitutions at residues 986 and 987, a “GSAS” substitution at furin cleavage site (residues 682–685) and C-terminal foldon trimerization motif, HRV3C protease recognition site, Twin-Strep-tag and octa-histidine tag cloned in mammalian vector pαH was used. The HexaPro construct was created by addition of four proline substitution (residues 817, 892, 899, 942) in 2P base construct as described previously.^18^ Plasmid encoding S protein was used to transiently transfect FreeStyle 293-F cells (Thermo Fisher) using polyethylenimine (PEI). Cells were maintained at a density of 0.2–3.0 × 10^6^ cells/mL at 37 °C, 8% CO_2_ and 125 rpm shaking in FreeStyle 293F media (Fisher Scientific). Transfection mix was prepared in Opti-MEM (Fisher Scientific) using two solutions, DNA (310 μg/l) and PEI max reagent (1 mg/mL, pH 7) in a ratio of 1:3 in 25 mL of Opti-MEM respectively, followed by addition of DNA solution to the PEI mix and incubated for 30 minutes at room temperature. Cells were transfected at a density of 1 × 10^6^ cells/mL and incubated at 37 °C, 8% CO_2_ and 125 rpm shaking. To elicit oligomannose-type glycans on S glycoprotein, 20 μM kifunensine was added at the time of transfection. Culture was harvested after 7 days post transfection and the media was separated from the cells by centrifugation at 4,000 rpm for 30 minutes.

The supernatant was filtered using 0.22 μm pore size filter (Merck) followed by S protein purification using 5 mL His Trap FF column connected to Akta Pure system (GE Healthcare). Prior to loading the sample, the column was washed with washing buffer (50 mM Na_2_PO_4_, 300 mM NaCl) at pH 7. The sample was loaded onto the column at a speed of 2 mL/min. The column was washed with washing buffer (10 column volumes) containing 50 mM imidazole and eluted in 3 column volumes of elution buffer (300 mM imidazole in washing buffer). The elution was concentrated by a Vivaspin column (100 kDa cut-off) to a volume of 1 mL and buffer exchanged to phosphate buffered saline (PBS). Further, purification of target S protein fraction was carried out using size-exclusion chromatography using a Superdex 200 16 600 column (GE healthcare). The target fraction was concentrated in 100 kDa vivaspin (GE healthcare) to a volume of 1 mL.

### Glycopeptide analysis by LC-MS

To perform the glycopeptide analysis using three protease enzymes, three 50 μg aliquots of SARS-COV-2 HexaPro were denatured for 1 h in 50 mM Tris/HCl, pH 8.0 containing 6 M of urea and 5 mM of dithiothreitol (DTT). Next, the S proteins were reduced and alkylated by adding 20 mM iodoacetamide (IAA) and incubated for 1 hr in the dark, followed by incubation with DTT to get rid of any residual IAA. The alkylated S proteins were buffer exchanged into 50 mM Tris/HCl, pH 8.0 using Vivaspin columns (3 kDa) and digested separately overnight using trypsin, chymotrypsin or alpha lytic protease (Mass Spectrometry Grade, Promega) at a ratio of 1:30 (w/w). The next day, the peptides were dried and extracted using C18 Zip-tip (MerckMilipore). The peptides were dried again, re-suspended in 0.1% formic acid and analyzed by nanoLC-ESI MS with an Easy-nLC 1200 (Thermo Fisher Scientific) system coupled to a Fusion mass spectrometer (Thermo Fisher Scientific) using higher energy collision-induced dissociation (HCD) fragmentation. Peptides were separated using an EasySpray PepMap RSLC C18 column (75 µm × 75 cm). A trapping column (PepMap 100 C18 3 μm (particle size), 75 μm × 2 cm) was used in line with the LC prior to separation with the analytical column. The LC conditions were as follows: 275 min linear gradient consisting of 0–32% acetonitrile in 0.1% formic acid over 240 min followed by 35 minutes of 80% acetonitrile in 0.1% formic acid. The flow rate was set to 300 nL/min. The spray voltage was set to 2.7 kV and the temperature of the heated capillary was set to 40 °C. The ion transfer tube temperature was set to 275 °C. The scan range was 400–1600 *m/z*. The HCD collision energy was set to 50%, appropriate for fragmentation of glycopeptide ions. Precursor and fragment detection were performed using an Orbitrap at a resolution MS1 = 100,000. MS2 = 30,000. The Automatic gain control (AGC) target for MS1 = 4e^5^ and MS2 = 5e^4^ and injection time: MS1 = 50 ms MS2 = 54 ms.

Glycopeptide fragmentation data were extracted from the raw file using Byonic™ and Byologic™ software (Version 3.5; Protein Metrics Inc.). The glycopeptide fragmentation data were evaluated manually for each glycopeptide; the peptide was scored as true-positive when the correct b and y fragment ions were observed along with oxonium ions corresponding to the glycan identified. The MS data was searched using the Protein Metrics N-glycan library, along with filtering of sulfated type glycans using wildcard search for sulfation (0, 1, 2, 3). Then the N309 mammalian glycan library was modified to include the sulfated glycans identified in previous searches which was then further used for determining glycan composition of 2P and HexaPro S protein. The relative amounts of each glycan at each site as well as the unoccupied proportion were determined by comparing the extracted chromatographic areas for different glycotypes with an identical peptide sequence. All charge states for a single glycopeptide were summed. The precursor mass tolerance was set at 4 ppm and 10 ppm for fragments. A 1% false discovery rate (FDR) was applied. The relative amounts of each glycan at each site as well as the unoccupied proportion were determined by comparing the extracted ion chromatographic areas for different glycopeptides with an identical peptide sequence. Glycans were categorized according to the composition detected. HexNAc(2)Hex(9–5) was classified as M9 to M5. HexNAc(3)Hex(5–6)Neu5Ac(0–4) was classified as Hybrid with HexNAc(3)Hex(5–6)Fuc(1)Neu5Ac(0–4) classified as Fhybrid. Complex-type glycans were classified according to the number of processed antenna and fucosylation. Complex glycans are categorized as HexNAc(3)(X), HexNAc(3)(F)(X), HexNAc(4)(X), HexNAc(4)(F)(X), HexNAc(5)(X), HexNAc(5)(F)(X), HexNAc(6+)(X) and HexNAc(6+)(F)(X). Any glycan containing at least one sialic acid was counted as sialylated. The quantifications of glycan compositions were represented as the mean of three biological replicates ± standard error of the mean.

### Determination of affinity using surface plasmon resonance (SPR)

Analysis of SARS-CoV-2 HexaPro binding with ACE2 protein was analyzed using a Biacore T200 (Cytiva/GE Healthcare). The proteins were buffer exchanged in the running buffer used for the SPR, HBS P+ (0.01 M HEPES pH 7.4, 0.15 M NaCl, 0.005% v/v Surfactant P20). Prior to injection of NiCl_2_, metallic contaminants were removed via a pulse of 350 mM ethylenediaminetetraacetic acid (EDTA) at a flow rate of 30 μL/min for 1 min. Followed by loading of Ni^2+^ at a flow rate of 10 μL/min for 1 min. SARS-CoV-2 S protein (50 μg/mL), ligand was injected at 10 μL/min for 240 s. Flow cell 2–1 was used in which one of the cells was used as a control for determination of non-specific binding to the chip. Control cycles were performed by flowing the analyte (ACE2 protein) over the control cell having absence of ligand (S protein); negligible binding was indicated. The analyte was serially diluted ranging from 200 nM to 3.125 nM in triplicated along with HBS P+ buffer only as a control and were injected at a flow rate of 50 μL/min. Association and dissociation time was set as 300 s and 600 s respectively. After each cycle, the chip was regenerated with a pulse EDTA (350 mM) for 1 min at a flow rate of 30 μL/min. The 1:1 binding model was used for fitting the resulting data using Biacore Evaluation Software (GE Healthcare) and subsequently fitted curves were used to calculate *K*_D_.

### Patient sample collection and ethical approval

Serological responses to 2P and HexaPro forms of SARS-CoV-2 S glycoprotein were analyzed in samples from acutely unwell intensive treatment unit (ITU) patients with SARS CoV-2, convalescent individuals who have had mild disease and normal control sera from pre-2019. We have previously shown that severity of disease affects the quantitation of antibody^35^ and so a spectrum of samples were used under ethics gained to aid assay development (NRES Committee West Midlands - South Birmingham 2002/201 Amendment Number 4, 24 April 2013) and from a Convalescent health care worker cohort (London - Camden & Kings Cross Research Ethics Committee 20/HRA/1817). Hospitalized subjects also provided nasopharyngeal swabs which were guanidine isothiocyanate inactivated, then analyzed by revers-transcriptase PCR directed against the SARS-CoV-2 ORF1ab and N genes (Viasure, CerTest Biotec). Pre-2019 negative control sera were obtained as part of a University of Birmingham study, reference ERN_16-178. All study participants gave written, informed consent and samples were fully anonymized.

### Serum ELISA methodology

All sera were obtained by centrifugation of whole blood at 3500 RPM for 5 mins, then stored at –20 °C until use. Antibodies to S glycoprotein were detected using an in-house developed, high-sensitivity ELISA, as previously described.^35^ In short, Nunc 96-well plates (ThermoFisher) or high binding plates (Corning) were coated with 2 μg/mL 2P or HexaPro or kifunensine HexaPro and blocked with 2% bovine serum albumin (Sigma Aldritch) (w/v) in PBS-T (Oxoid phosphate buffered saline with 0.1% Tween-20, Sigma Aldritch). Serum was initially diluted 1:40 in PBS-T and then serially diluted. Secondary antibodies (combined horse radish peroxidase-conjugated mouse anti-human IgG, A and M monoclonal antibodies) were diluted in PBS-T (anti–IgG R-10 1:8,000, anti–IgA MG4.156 1:4,000, and anti-IgM AF6 1:2,000; Abingdon Health). Signal was developed using TMB-Core (Bio-Rad) for between 6 and 12 minutes then stopped with 0.2 M H_2_SO_4_ (Sigma-Aldrich). Optical density (OD) at 450 nm was detected using the Dynex DSX automated liquid handler (Dynex Technologies, USA). Signal:noise ratio (S:N ratio) was calculated by dividing the individual OD values from PCR+ serum samples (signal) by the average OD from the pre-2019 negative controls (noise). Statistical significance was assessed using a RM 2-way ANOVA with Geisser-Greenhouse correction, followed by Sidak’s multiple comparisons test, using Graphpad Prism version 8.

### Integrative modelling and molecular dynamics simulation

Three S protein models were built using Modeller version 9.21^80^: (i) 2P with one RBD in the “up” conformation, (ii) HexaPro with one RBD in the “up” conformation, and iii) HexaPro with two RBDs in the “up” conformation. For 2P, the S protein ectodomain (ECD) was modelled using the cryo-EM structure of SARS-CoV-2 2P S ECD in the open state (PDB: 6VSB).^17^ The ECD of the one-RBD-up HexaPro was modelled using the cryo-EM structure of SARS-CoV-2 HexaPro S with one RBD up (PDB: 6XKL).^18^ For the two-RBD-up ECD, we performed molecular dynamics flexible fitting (MDFF),^81^ whereby the atomic coordinates of the HexaPro one-RBD-up structure was fitted to the electron density map of HexaPro two-RBD-up (EMDB: EMD-22222).^18^ The initial structure was prepared in VMD^82^ and MDFF was performed in vacuum using NAMD version 2.11^83^ with the CHARMM36 force field.^84^ The MDFF simulation was performed until convergence using a range of scaling factors from 0.3 to 40, with secondary structural and domain restraints applied to the protein. The stalk and the transmembrane (TM) domain of all three models were built using the NMR structure of SARS-CoV HR2 domain (PDB: 2FXP)^85^ and the NMR structure of HIV-1 gp-41 TM domain (PDB: 5JYN),^43^ respectively, while missing loops in the NTD and the C terminus of the ECD were modelled based on a higher resolution cryo-EM structure of S protein in the closed state (PDB: 6XR8).^86^ The same modelling protocol previously described to build a full-length model of the wild-type SARS-CoV-2 S protein was used.^87^ The aim of the modelling and MD simulation study was to measure the accessible surface area (ASA) for each unprocessed glycosylation sites, in order to ascertain likely accessibility to glycan processing enzymes, as previously described in Allen et al.^52^ Man-9 represents the primary substrate for glycan processing enzymes; as such Man-9 glycans were added to all 22 glycosylation sites using CHARMM-GUI Glycan Reader and Modeller.^88^ The glycosylated S protein models were then embedded into a pre-equilibrated model of the endoplasmic reticulum-Golgi intermediate compartment (ERGIC) membrane^89^ built using CHARMM-GUI Membrane Builder.^90^ The system was solvated with TIP3P water molecules and neutralized with 0.15 M NaCl salt. Stepwise energy minimization and equilibration simulations with decreasing amount of positional and dihedral restraints were conducted following the standard CHARMM-GUI protocols.^91^ For each S protein model, three replicates of 200 ns production simulation were performed. The Nosé-Hoover thermostat was used to maintain the temperature at 310 K,^92^, ^93^ while a semi-isotropic coupling to the Parrinello-Rahman barostat was used to maintain the pressure at 1 atm.^94^ The electrostatic interactions were calculated using the smooth particle mesh Ewald method with a real-space cut-off of 1.2 nm,^95^ and the van der Waals interactions were truncated at 1.2 nm with a force switch smoothing applied between 1.0 and 1.2 nm. The simulations employed a 2 fs integration time step with the LINCS algorithm applied on all covalent bonds involving hydrogen atoms.^96^ All simulations were run using GROMACS 2018^97^ and the CHARMM36 force field.^84^ ASA calculation was performed using the GROMACS tool *gmx sasa*, based on the last 50 ns of each trajectory. Comparison of ASA values between replica simulations showed overlapping values within error bars at most sites (Figure S6), and hence an average value was calculated to represent each glycosylation site.

### Glycan analysis by HILIC-UPLC

Gel bands corresponding to the HexaPro S and glycan engineered HexaPro S protein (kifunensine-treated) were excised and washed three times with alternating 1 ml acetonitrile and water, incubating and shaking for 5 minutes following addition of each wash solution. All liquid was removed following the final wash stages and N-linked glycans were released in-gel using PNGaseF, (2 µg enzyme in 100 µL H_2_O) (New England Biolabs) at 37 °C overnight. Following digestion, the liquid was removed from the gel bands and placed into a separate Eppendorf. The gel bands were then washed twice with 100 µl MilliQ H_2_O and this was pooled with the original solution. The extracted glycans were then dried completely in a speed vac at 30 °C.

The released glycans were subsequently fluorescently labelled with procainamide using 110 mg/ml procainamide and 60 mg/ml sodium cyanoborohydride in a buffer consisting of 70% DMSO, 30% acetic acid. For each sample, 100 µl of labelling mixture was added. Labelling was performed at 60 °C for 2 hours. Excess label and PNGaseF were removed using Spe-ed Amide-2 cartridges (Applied Separations). First, the cartridges were equilibrated sequentially with 1 ml acetonitrile, water and acetonitrile again. Then 1 ml of 95% acetonitrile was added to the procainamide-released glycan mixture and applied to the cartridge, allowing the cartridge to drain by gravity flow. After the mixture has emptied the cartridge, two washes using 97% acetonitrile were performed. To elute the labelled glycans 1 ml HPLC grade water was added to the cartridges and the elution collected. The elution was then dried completely using a speed vac, before resuspending in 24 µl of 50 mM ammonium formate.

A 6 µl aliquot of the resuspended glycans were mixed with 24 µl of acetonitrile and analysed on a Waters Acquity H-Class UPLC instrument with a Glycan BEH Amide column (2.1 mm × 150 mm, 1.7 μM, Waters), with an injection volume of 10 µl. A gradient of two buffers; 50 mM ammonium formate (buffer A) and acetonitrile (buffer B) was used for optimal separation. Gradient conditions were as follows: initial conditions, 0.5 ml/min 22% buffer A, increasing buffer A concentration to 44.1% over 57.75 minutes. Following this the concentration of buffer A was increase to 100% at 59.25 minutes and held there until 66.75 minutes and the flow rate was dropped to 0.25 ml/min, to fully elute from the column. Finally, the %A was reduced to 20% to prepare for subsequent runs. Wavelengths used for detection of the procainamide label were: excitation 310 nm, emission 370 nm. Data were processed using Empower 3 software (Waters, Manchester, UK). The relative abundance of oligomannose-type glycans was measured by digestion with Endoglycosidase H (per sample in 20 µl volume) (Endo H; New England Biolabs). A 6 µl aliquot of labelled glycans was combined with 1 µg endoH to a final volume of 20 µl. Digestion was performed overnight at 37 °C.

Digested glycans were cleaned using a 96-well PVDF protein-binding membrane (Millipore) attached to a vacuum manifold. Prior to application to the membrane, 100 µl HPLC-grade H_2_O was added to each sample. Following equilibration with 150 µl ethanol, and 2 × 150 µl HPLC-grade H_2_O, the sample was added to the 96-well plate and the flow-through was collected in a 96-well collection plate. Each well was then washed twice with HPLC-grade H_2_O to a final elution volume of 300 µl. The elution was then dried completely at 30 °C. Prior to analysis the sample was resuspended in 6 µl ammonium formate and 24 µl acetonitrile and analysed as detailed above.

#### CRediT authorship contribution statement

**Himanshi Chawla:** Conceptualization, Investigation, Formal analysis, Writing – original draft, Writing – original draft, Writing – review & editing. **Sian E. Jossi:** Investigation, Formal analysis, Writing – review & editing. **Sian E. Faustini:** Investigation, Formal analysis, Writing – review & editing. **Firdaus Samsudin:** Investigation, Formal analysis, Writing – review & editing. **Joel D. Allen:** Investigation, Writing – review & editing. **Yasunori Watanabe:** Investigation, Writing – review & editing. **Maddy L. Newby:** Investigation, Writing – review & editing. **Edith Marcial-Juárez:** Investigation, Writing – review & editing. **Rachel E. Lamerton:** Investigation, Writing – review & editing. **Jason S. McLellan:** Conceptualization, Supervision, Funding acquisition, Writing – review & editing. **Peter J. Bond:** Conceptualization, Supervision, Funding acquisition, Writing – review & editing. **Alex G. Richter:** Conceptualization, Supervision, Funding acquisition, Writing – review & editing. **Adam F. Cunningham:** Conceptualization, Supervision, Funding acquisition, Writing – review & editing. **Max Crispin:** Conceptualization, Funding acquisition, Supervision, Writing – original draft, Writing – review & editing.
