# Supplementary material for: Glycosylation and Serological Reactivity of an Expression-enhanced SARS-CoV-2 Viral Spike Mimetic
Source: J Mol Biol. 2022 Jan 30;434(2):167332. doi: 10.1016/j.jmb.2021.167332 (PMC8550889; doi:10.1016/j.jmb.2021.167332)
Supplement: Supplementary data 1 [file mmc1.docx]

**Supporting Information for:**

**Glycosylation and serological reactivity of an expression-enhanced SARS-CoV-2 viral spike mimetic**

Himanshi Chawla^1^, Sian E. Jossi^2^, Sian E. Faustini^2^, Firdaus Samsudin^3^, Joel D. Allen^1^, Yasunori Watanabe^1,4^, Maddy L. Newby^1^, Edith Marcial-Juárez^2^, Rachel E. Lamerton^2^, Jason S. McLellan^5^, Peter J. Bond^3,6^, Alex G. Richter^2^, Adam F. Cunningham^2^, Max Crispin^1*^

^1^ School of Biological Sciences, University of Southampton, Southampton, SO17 1BJ, UK.

^2^ Institute of Immunology and Immunotherapy, University of Birmingham, Birmingham, B15 2TT, UK.

^3^ Bioinformatics Institute, Agency for Science, Technology and Research (A*STAR), Singapore, 138671, Singapore.

^4^ Oxford Glycobiology Institute, Department of Biochemistry, University of Oxford, South Parks Road, Oxford, OX1 3QU, UK.

^5^ Department of Molecular Biosciences, The University of Texas at Austin, Austin, TX 78712, USA.

^6^ Department of Biological Sciences, National University of Singapore, Singapore 117543, Singapore

* To whom correspondence may be addressed. Email: max.crispin@soton.ac.uk

**Supplementary Table S1**. **Abundance of glycoform observed across SARS-CoV-2 HexaPro S protein.** The left-hand table represents the average of glycan composition at each N-linked glycan site obtained from the reported values of three biological replicates. The right-hand table is representing the global averages of glycan compositions at all the N-linked glycan sites.

**Supplementary Table S2**. **Glycoform abundances observed across SARS-CoV-2 2P S protein.** The table represents the glycan composition at each N-linked glycan site. The data is reanalysed to include sulfated glycans from the previously published analysis [1]. The left-hand table is representing the average of reported values from three biological replicates. The global averages are shown in the right-hand table.

**Supplementary Table S3. Comparison of composition of glycoforms across variants of SARS-CoV-2 S protein, HexaPro and 2P.** The table illustrates the difference between HexaPro and 2P at each N-linked glycan site from the average of three biological repeats of each variant. The top left-panel represents the average of the glycan composition difference between HexaPro and 2P at individual N-linked glycan sites. The top right-panel represents the global average difference between HexaPro and 2P at all N-linked glycan sites. The lower left-panel represents the sum of M9-M5 as mannose, sum of hybrid and Fhybrid as Hybrid, sum of HexNAc(3)(X) to HexNAc(6+)(F)(X) as complex, and unoccupied across all N-glycan sites. The lower-right panel represents the sum of global average composition difference between HexaPro and 2P. The increase in abundance of glycan composition in HexaPro is represented in range from yellow to green whereas the increase in abundance of glycan composition in 2P is shown in range from yellow to red”.

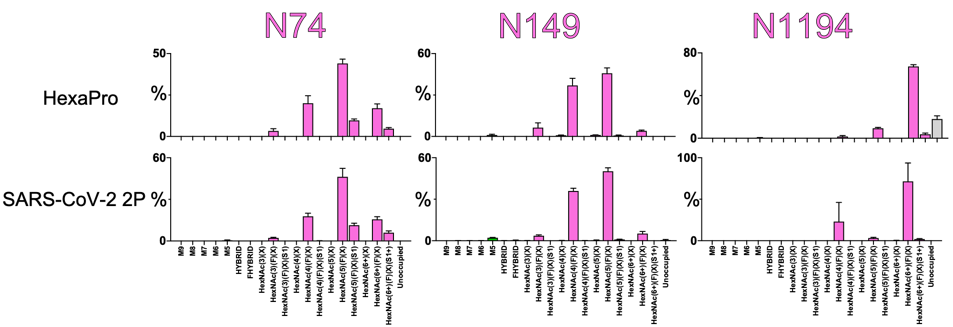


**Supplementary Figure S1. Representation of extensive site-specific analysis of N-linked glycosylation sites of SARS-CoV-2 2P and HexaPro.** The recombinant proteins were digested using multiple proteases, analysed by LC-MS, and the resulting data was searched using library containing sulfo groups. The bar graph illustrates the values from three biological repeats. The sites represented here are the only ones which has shown the sulfation in the analysis. The abbreviation used here are M, mannose; X, Hex & Neu5Ac; F, Fucose, S, sulfation.

**Supplementary Figure S2:** **Comparison of ASA values between two-RBD-up HexaPro and 2P S protein.** ΔASA is calculated as the arithmetic difference between the ASA values (ASA_2P_ - ASA_HexaPro_) averaged over the last 50 ns of the simulations and across three replicate simulations. A positive value thus represents a lower accessibility in HexaPro, therefore a potential increase in oligomannose-type glycans, and vice versa. The error bars show standard deviations along the trajectories and across repeat simulation.

**Supplementary Figure S3:** **Comparison of glycan N165 from simulations of two-RBD-up HexaPro and 2P S protein variants.** Snapshot at the beginning (left) and at the end (middle) of the HexaPro simulation, highlighting N165 glycan from chain A. Snapshot at the end of the 2P simulation (right) showing the same glycans. Protein is shown in surface representation and coloured pink (chain A), cyan (chain B), or green (chain C), whilst the glycan is shown in stick representation and coloured in orange.

**Supplementary Figure S4:** **Comparison of protein dynamics between HexaPro and 2P.** (A) Root mean square fluctuation (RMSF) of each residue in the ectodomain (ECD) of HexaPro and 2P from 200 ns simulations. The position of the RBD is highlighted in grey and the positions of six proline mutations in HexaPro are marked with black circles and labelled. Thick lines represent average values from three repeat simulations and the shaded areas indicate standard deviation. (B) The first principal motion of all backbone atoms on the ECD of chain C of HexaPro two-RBD-up (left) and 2P (right) as determined by principal component analysis.


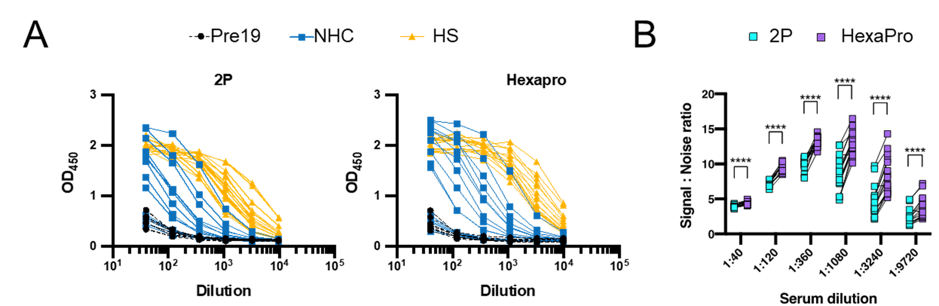


**Supplementary Figure S5.** **Antibody responses detected in an ELISA using 2P or HexaPro Spike as the target antigen**. A) Absorbance values of sera serially diluted 3-fold from Pre19 (black circles, dashed lines), NHC (blue squares) or HS (yellow triangles) donors. B) Signal: Noise ratio calculated using individual PCR+ HS sera OD values as signal and the mean Pre19 sera as noise at multiple serum dilutions. Each point represents an individual signal: noise ratio from one serum, with a line connecting the same serum sample tested against 2P (cyan) or HexaPro (violet).

**Supplementary Figure S6. Comparison of ASA values from replicate simulations.** Example ASA values calculated from three independent 200 ns simulations. Data taken from the last 50 ns of HexaPro two-RBD-up simulations from S protein chain A. Average values are shown with standard deviations along the trajectories depicted as error bars.

References:

[1] Y. Watanabe, J.D. Allen, D. Wrapp, J.S. McLellan, M. Crispin, Site-specific glycan analysis of the SARS-CoV-2 spike, Science. 369 (2020) 330–333. https://doi.org/10.1126/science.abb9983.
